# Immunohistochemical Expression of Calretinin in Dentigerous Cyst Transforming Into Unicystic Ameloblastoma: A Case Report

**DOI:** 10.7759/cureus.68938

**Published:** 2024-09-08

**Authors:** Anchal Varshney, Manish Bhargava, Pratijya Raj

**Affiliations:** 1 Department of Oral Pathology and Microbiology, Manav Rachna Dental College, Faridabad, IND; 2 Department of Oral Pathology, Manav Rachna Dental College, Faridabad, IND

**Keywords:** calretinin, dentigerous cyst, immunohistochemical marker, luminal proliferation, unicystic ameloblastoma

## Abstract

The term "unicystic ameloblastoma" describes cystic lesions that exhibit radiographic, clinical, or gross characteristics of a jaw cyst. However, histologic examination reveals a typical ameloblastomatous epithelium lining the cyst cavity, with or without luminal and/or mural tumor proliferation. Unicystic ameloblastoma is a less prevalent kind of ameloblastoma. Among the odontogenic cysts, neoplastic transformation is highest in odontogenic keratocysts (OKCs) and dentigerous cysts (DCs). Calretinin is considered a specific immunohistochemical marker for neoplastic ameloblastic epithelium and serves as a diagnostic tool for differentiating odontogenic cystic lesions from ameloblastic tumors. This paper illustrates a case of a DC transforming into unicystic ameloblastoma in a 22-year-old male patient using the immunohistochemical expression of calretinin.

## Introduction

Benign tumors called ameloblastomas are significant because they can grow to large sizes and cause deformities in the bone. Generally, they are divided into peripheral, malignant, multicystic, and unicystic subtypes [1]. A less common form of ameloblastoma, unicystic ameloblastoma refers to cystic lesions that exhibit odontogenic cystic characteristics in terms of clinical presentation and radiographic analysis but which, upon microscopic examination, show a typical ameloblastomatous epithelium, either with or without luminal and/or mural tumor growth, lining a section of the cyst cavity [2]. Few odontogenic cysts, such as dentigerous cysts (DCs), glandular odontogenic cysts, calcifying odontogenic cysts, radicular cysts, and odontogenic keratocysts (OKCs) have the potential to develop into neoplasm. The neoplastic transformation rate in odontogenic cysts is higher in DC and OKC [3]. This paper focuses on the importance of microscopic examination and immunohistochemistry with calretinin biomarkers in DCs transforming into unicystic ameloblastoma.

## Case presentation

**Figure 1:**
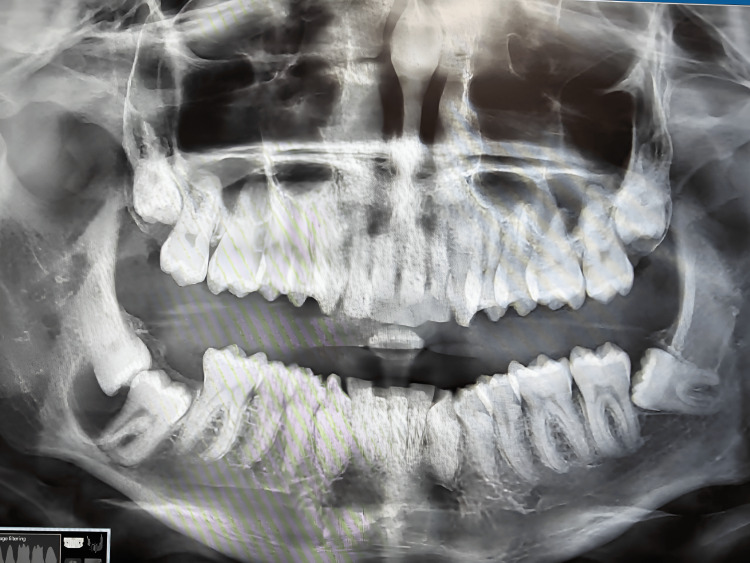
OPG revealed a unilocular radiolucency surrounding the crown of the impacted 48 OPG: Orthopantomogram

A 22-year-old male reported a chief complaint of missing teeth in the right back tooth region. There was no visible edema in the right lower posterior region of the face during extraoral examination. An intraoral examination found a very small, painless, diffuse enlargement in the right mandibular posterior region, with no facial asymmetry. The swelling was firm and the overlying mucosa appeared normal. Teeth 47 and 48 were absent, and tooth 46 was vital. On radiographic examination, the orthopantomogram (OPG) revealed a unilocular radiolucency surrounding the crown of the impacted tooth 48. Tooth 47 appeared to be submerged (Figure 1).

**Figure 2:**
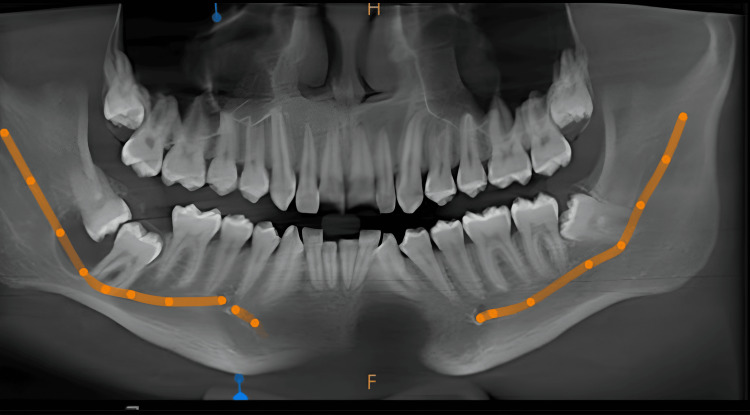
CBCT showing a well-defined unilocular radiolucency surrounding the crown of horizontally impacted 48 CBCT: Cone beam computed tomography

**Figure 3:**
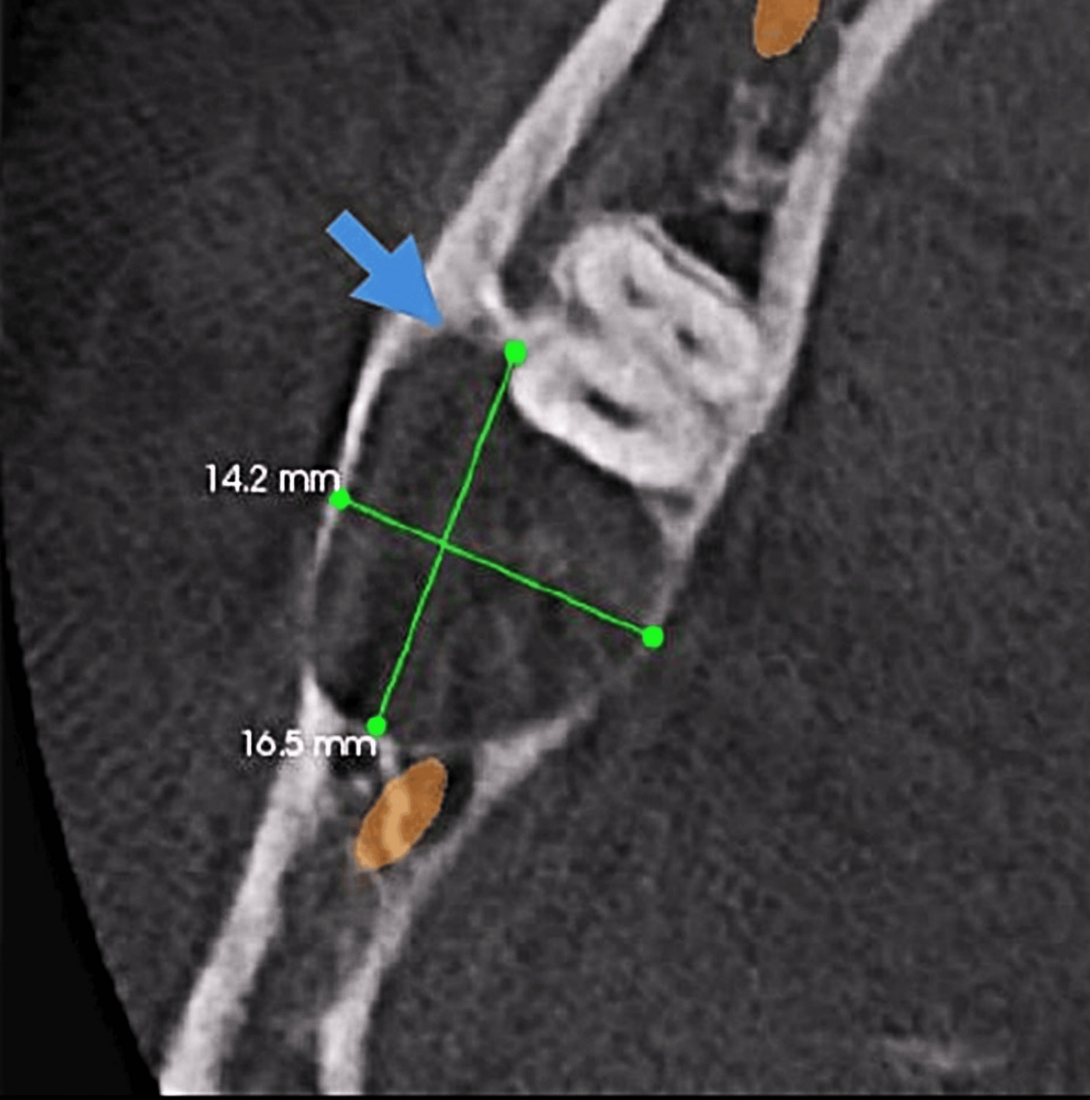
Axial view of CBCT revealed a well-defined solitary radiolucent lesion of approximately 16.5 × 20.7 × 14.2 mm (AP × SI × BL) with respect to the right posterior mandibular anterior ramus region, extending anteroposteriorly from CEJ of 48 to distal aspect of 47, and superoinferiorly from crestal bone to approximately 8.8 mm from inferior border of mandible. CBCT: Cone beam computed tomography; AP: Anteroposterior; SI: Superoinferior; BL: Buccolingual; CEJ: Cementoenamel junction

The cone beam computed tomography (CBCT) also revealed horizontally impacted tooth 48. A well-defined solitary radiolucent lesion of size approximately 16.5 × 20.7 × 14.2 mm (anteroposterior × superoinferior × buccolingual) was noted with respect to the right posterior mandibular anterior ramus region extending anterior-posteriorly from the cementoenamel junction (CEJ) of tooth 48 to the distal aspect of tooth 47 and superior-inferiorly from the crestal bone to approximately 8.8 mm from the inferior border of the mandible. Apically displaced right inferior alveolar nerve canal (IANC) was noted. Thinning/Discontinuity of both buccal and lingual cortical plate with respect to tooth 48 was seen. No signs of root resorption or fracture were noted with respect to tooth 47. Infra-erupted tooth 47 and approximation of right IANC with both mesial and distal roots of tooth 47 was also seen (Figures 2-3).

**Figure 4:**
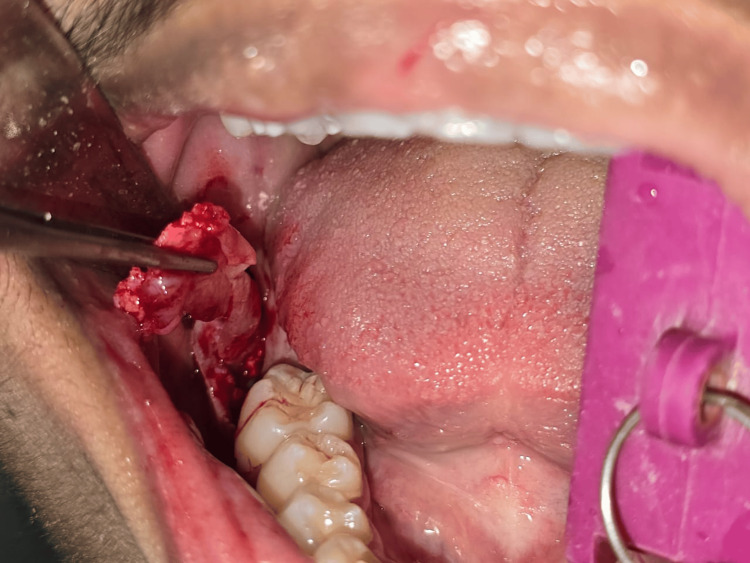
Enucleation of cystic lesion under local anesthesia

**Figure 5:**
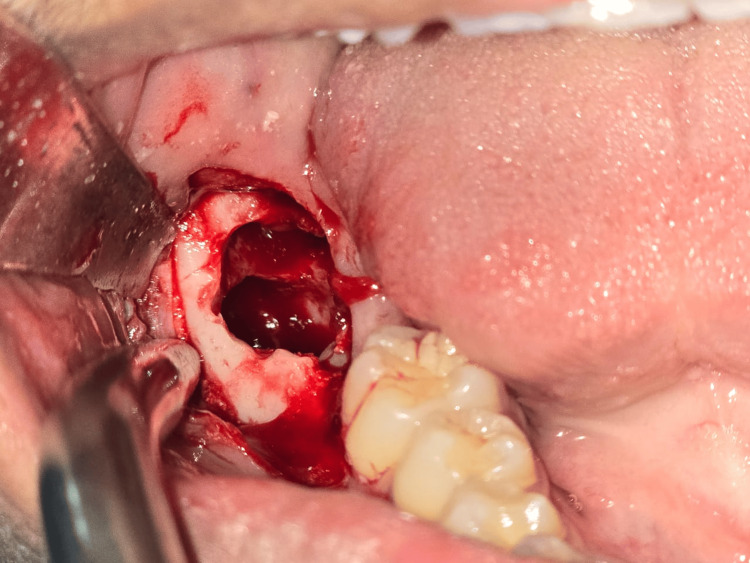
Cystic cavity after enucleation

Enucleation of the cystic lesion was done *in toto*, and the cavity irrigation was done using 0.9% normal saline after extraction of tooth 48 under local anesthesia (Figures 4-5). Primary closure of the cavity was done using 3-0 silk. A provisional diagnosis of DC was made based on the radiological and clinical features. The sample was sent to the Department of Oral Pathology for histopathological examination.

**Figure 6:**
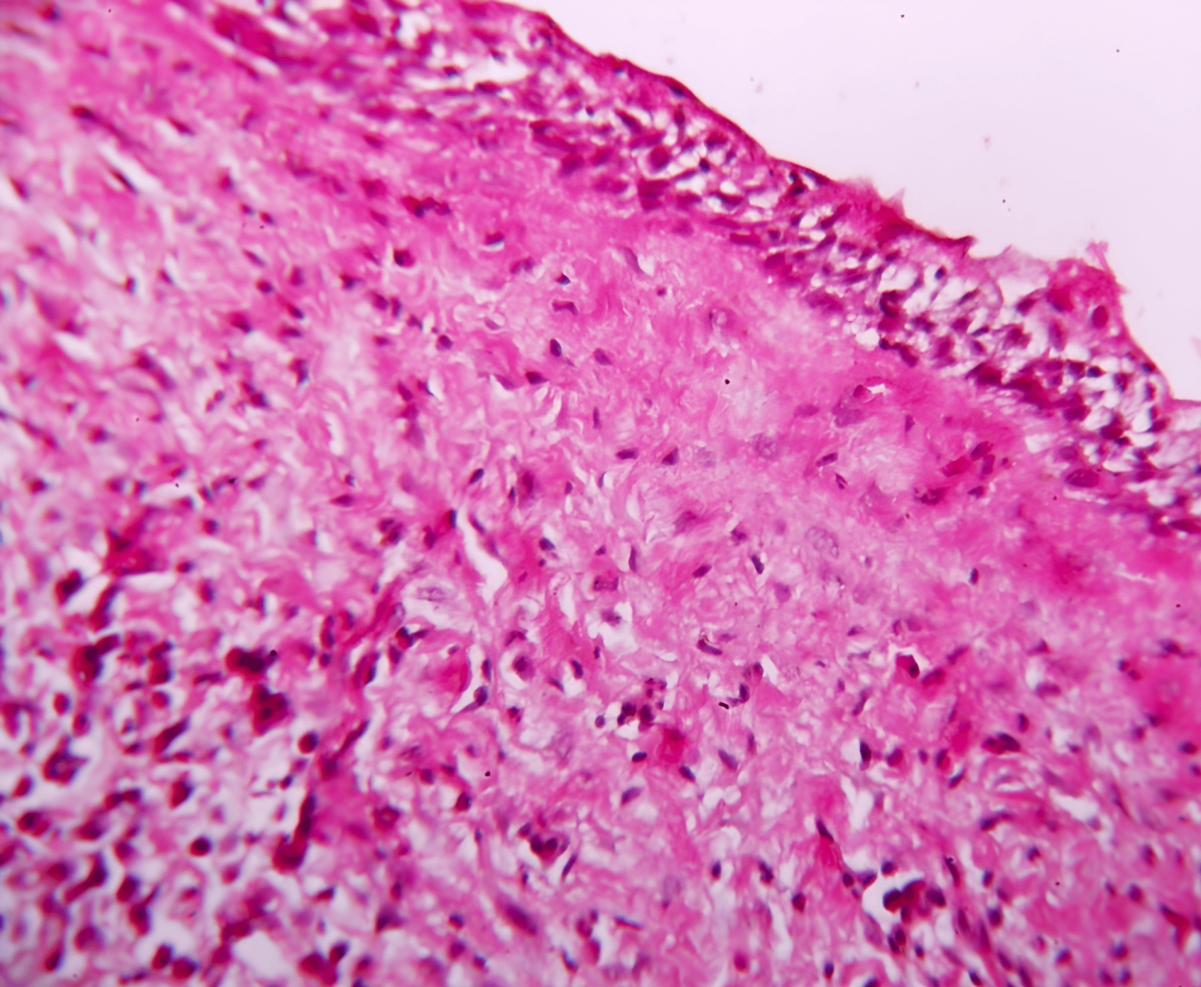
Photomicrograph magnification (40x) of suprabasilar epithelial cells, loosely cohesive and resembling stellate reticulum-like cells

The hematoxylin and eosin (H&E) stained tissue section revealed a cystic lumen lined by a thin layer of Odontogenic epithelium. A basal layer of columnar cells with hyperchromatic nuclei that displayed reversed polarity was seen in the epithelium. The suprabasilar epithelial cells were loosely cohesive and resembled stellate reticulum-like cells. The underlying connective tissue was fibrocellular with infiltration of chronic inflammatory cells and blood vessels of varying size and shape (Figure 6).

**Figure 7:**
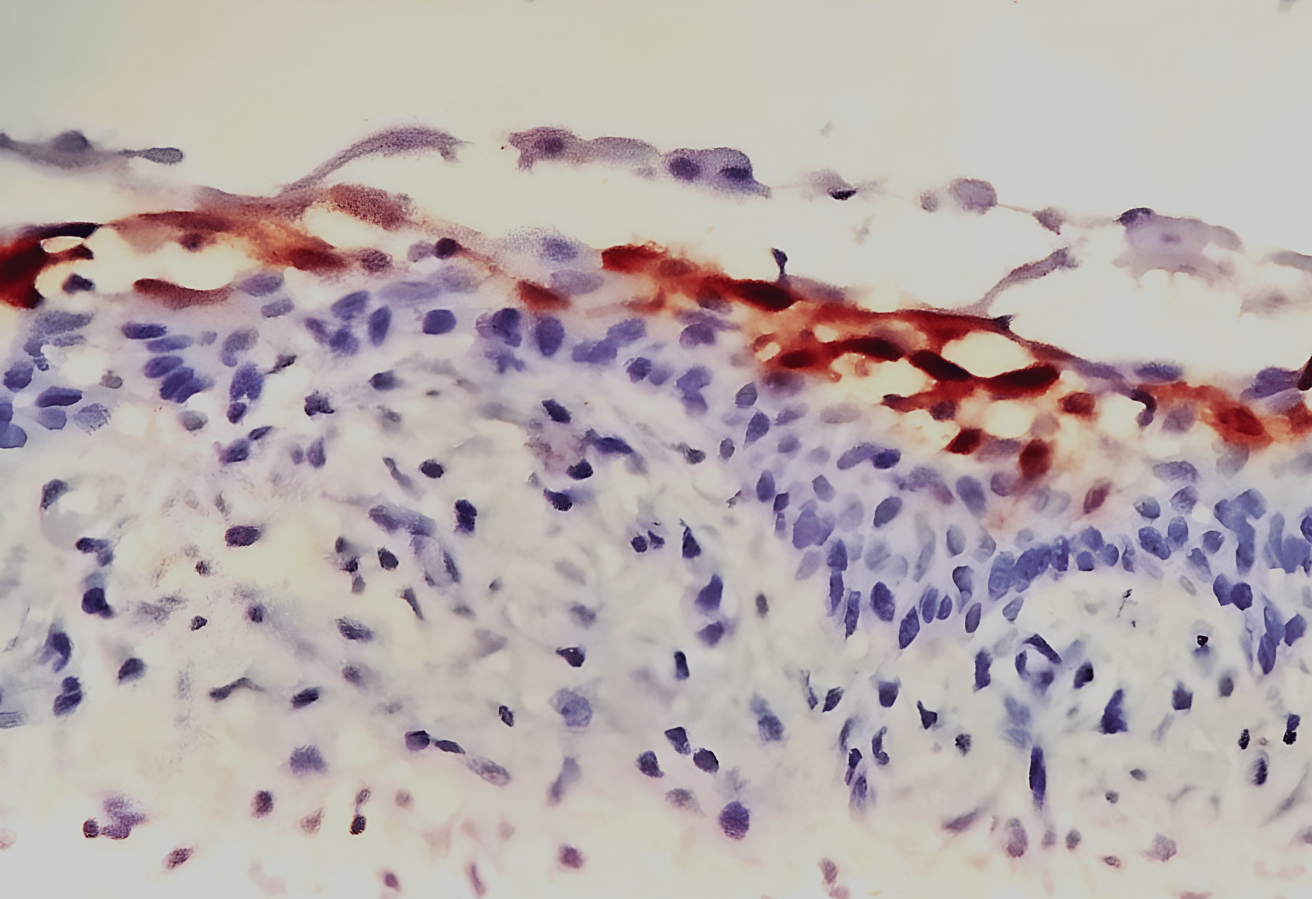
Positive calretinin staining in the lining of unicystic ameloblastoma magnification (40x)

The expression of calretinin was also examined because it is an immunohistochemical marker specific to the neoplastic ameloblastic epithelium and plays a role in the change from odontogenic cyst epithelial lining to ameloblastomatous epithelium. Therefore, it can be a diagnostic tool to distinguish ameloblastic tumors from cystic odontogenic lesions. The section treated with immunohistochemistry showed positive expression of calretinin. (Figure 7).

Based on the above features, the final diagnosis was unicystic ameloblastoma. The healing after surgery was uneventful.

## Discussion

Robinson and Martinez were the first to describe unicystic ameloblastoma as a type of ameloblastoma. Male patients make up most of the patient population, with an average age of 25.5 years and 46% of cases occurring in the second decade of life. In the mandible, typically in the posterior area, are more than 90% of them. The lesion is usually painless, though a severe lesion may cause the jaws to enlarge without any pain [4]. Similar findings were reported in this case of the 22-year-old male patient in the posterior region of the mandible. Peter reports that of all ameloblastoma subtypes, the incidence of unicystic ameloblastoma varies from 5% to 22% [5]. There is a lack of available data regarding the incidence and prevalence of UA. Whether unicystic ameloblastoma originates in an already-existing cyst or develops de novo has been the subject of numerous discussions. Three pathogenic pathways were hypothesized to explain the evolution of unicystic ameloblastoma: reduced enamel epithelium, DC, and cystic degeneration of solid ameloblastoma. The focal portion of the lining of cystic tumors often consists of a thin, epithelium that resembles the DC lining and frequently involves an impacted tooth [6]. 

In the present case, the second hypothesis that the association between the cystic tumor and an impacted tooth, as well as the presence of non-specific thin epithelial lining in localized sections of the tumor, supports the theory that the cystic tumor arose from an underlying DC. The most common radiological patterns for unicystic ameloblastoma were determined by Eversole et al. to be unilocular, scalloped, pericoronal, interradicular, or periapical expansile radiolucencies [7].

Additionally, a distinct unilocular radiolucency that encircles the impacted tooth's crown was visible in the present case. Microscopically, the odontogenic epithelium lining the cyst lumen is seen in UA. Its thickness varies, ranging from a few layers to multiple cell layers, and it exhibits the typical cytomorphologic features of ameloblastoma as described by Vickers and Gorlin. These features include a basal cell layer made up of columnar cells that exhibit hyperchromatism, palisaded nuclei, reverse polarity, and subnuclear vacuoles. Also visible is a thin layer of cells that resemble stellate reticulum on top [8]. Similar findings were also seen in the present case. A characteristic ameloblastomatous epithelium lining, on the histologic investigation of cystic lesions that exhibit clinical, radiological, or gross signs of a jaw cyst. These lesions are referred to as UAs [6]. Because of the potential for misdiagnosing situations such as DCs, it is advisable to use advanced diagnostic techniques such as immunohistochemistry. It has been proposed that the calcium-binding protein calretinin serves as a unique immunohistochemistry marker for ameloblastic tissues. In this case, the positive expression of calretinin was consistent with several findings reported in the literature [9]. Anandani et al. observed calretinin expression in 50% of cases of unicystic ameloblastomas [10]. However, it is less than the numbers published by Altini et al. who found that 81.5% and 80%, respectively, of unicystic ameloblastomas had positive calretinin expression [11]. Furthermore, 100% of unicystic ameloblastomas had a positive expression of calretinin, according to studies by DeVilliers et al. and Sundaragiri et al [12,13].

The biological behavior of unicystic ameloblastoma is arguably the most significant factor to account for. Many agree that these lesions are not as aggressive as their solid or multicystic counterparts and that curettage or enucleation would be the best course of action. Gupta has highlighted the biological differences between lesions that are just cystic or show intraluminal proliferation and those in which the epithelium has penetrated the fibrous wall and can permeate the cancellous bone. Compared to the mandible, the maxilla exhibits greater aggression. Ameloblastomas can also develop into malignant entities, such as ameloblastic carcinoma and metastatic ameloblastoma, with a mere 2% prevalence rate [1]. Compared to other ameloblastoma variants such as follicular type, which exhibits a recurrence rate of approximately 30%, unicystic ameloblastoma is reported to have a low recurrence rate. Following conservative surgical therapy (curettage or enucleation), the recurrence rate for UAs is typically stated to be between 10-20%, with an average of less than 25%. Long-term follow-up is essential regardless of the surgical strategy the surgeon chooses to use, since unicystic ameloblastoma recurrence may occur years later [14].

## Conclusions

DCs are frequently associated with a permanent tooth that is not yet erupting. Untreated DCs can infrequently develop into odontogenic tumors such as ameloblastoma, as well as malignancies such as mucoepidermoid carcinoma and oral squamous cell carcinoma. Pathologists and physicians need to determine whether an ameloblastomatous alteration is occurring in a DC to make an accurate diagnosis and choose the best course of treatment. For this reason, it may be useful to identify odontogenic cystic transition to malignancies by using particular immunohistochemistry markers such as calretinin for neoplastic ameloblastic epithelium.
